# Letter to the editor regarding “Chemical disinfection in healthcare settings: critical aspects for the development of global strategies”

**DOI:** 10.3205/dgkh000393

**Published:** 2021-06-24

**Authors:** Roald Papke

**Affiliations:** 1 Sana Klinikum Lichtenberg, Berlin, Germany

## Letter to the editor

In the publication by Exner et al. [1], it states “Alcohol-based handrubs, containing ethanol or 1-propanol or 2-propanol as their main active substances are the gold standard for handrubs in Europe. For example, the German KRINKO recommends the use of alcohol-based products without any other additives such as chlorhexidine (CHG) or mecetronium ethylsulfate. Concern as to resistance development of certain bacterial strains to chlorhexidine is increasing, e.g., CHG resistance may be detected in multi-resistant isolates such as extremely drug-resistant Klebsiella pneumoniae [2], [3], [4], [5], [6].”

In the cited KRINKO recommendation [4] sustained effective additives in alcohol-based hand disinfectants are not recommended, and the following antiseptic agents are given exemplary: chlorhexidine digluconate, octenidine hydrochloride, polihexanide, triclosan, quaternary ammonium compounds, ampholytes and phenol derivatives. In this context, allow me to ask whether mecetronium ethylilsulfate can be used as an active ingredient. 

## Notes

### Competing interests

The author declares that he has no competing interests.
